# Lymphocyte to C-reactive protein ratio and improved prognostication of renal cell carcinoma: machine learning-based analysis

**DOI:** 10.3389/fonc.2026.1907942

**Published:** 2026-10-13

**Authors:** Melis Guer, Cesare Saitta, Viraj Master, Hajime Tanaka, Dattatraya Patil, Margaret F. Meagher, Dhruv Puri, Kit Yuen, Natalie Birouty, Giacomo Musso, Mai Dabbas, Aaron Ahdoot, Masaki Kobayashi, Mimi Nguyen, Alexander Hill, Omer Baker, Shohei Fukuda, Mohamed Elshaer, Michael A. Liss, Edward Cherullo, Yasuhisa Fujii, Ithaar H. Derweesh

**Affiliations:** 1 Department of Urology, University Hospital Tübingen, Tübingen, Germany; 2 Department of Urology, UC San Diego School of Medicine, La Jolla, CA, United States; 3 Department of Urology, Emory University School of Medicine, Atlanta, GA, United States; 4 Department of Urology, Institute of Science Tokyo, Tokyo, Japan; 5 Department of Urology, RUSH-MD Anderson Cancer Center, Rush University Medical Center, Chicago, IL, United States

**Keywords:** carcinoma, renal cell, C-reactive protein, lymphocyte count, nephrectomy, survival analysis, UISS

## Abstract

**Purpose:**

To investigate utility of a novel ratio, lymphocyte to C-reactive protein ratio (LCR), for improved prognostic stratification in RCC.

**Methods:**

We conducted a retrospective machine learning–based analysis in a multicenter RCC cohort who underwent surgery (Radical or Partial Nephrectomy). Cut-off for “low” or “high” LCR was delineated by receiver operating characteristics/area under curve (ROC/AUC). Multivariable Analysis was conducted for predictors of all-cause mortality (ACM) and cancer-specific mortality (CSM). LCR, along with UCLA Integrated Staging System (UISS) factors, were included into MVA model to evaluate CSM. Employing Classification and Regression Tree (CART), we developed a decision tree based on hazard ratios (HR). The predictive performance was calculated using ROC/AUC.

**Results:**

We analyzed 2071 patients [low LCR n=598/high LCR n=1473; cut-off=237.87 (AUC = 0.80)], median follow-up 60 months). Low LCR is an independent risk factor for ACM (HR = 2.75, p<0.001) and CSM (HR = 2.70, p<0.001). Including LCR (HR = 2.60, p<0.001), tumor stage (Stage II: HR = 6.7, Stage III: HR = 6.5, Stage IV: HR = 26.8, p<0.001) nuclear grade (HR = 3.0, p<0.001) and ECOG (HR = 1.6, p=0.002) in the CART, patients were re-stratified [(HR<1.0 (low-risk), HR = 1.0-2.0 (intermediate-risk), HR>2.0 (high-risk)]. AUC of 0.91 was revealed for the modified UISS model (AUC = 0.85 in the classic UISS model). Our findings are limited by retrospective design.

**Conclusion:**

This study provides evidence supporting the prognostic significance of LCR in RCC. While low LCR was associated with poorer survival outcomes, incorporating LCR into the UISS model enhanced prognostic stratification in RCC patients. Further investigation is requisite to validate our findings.

## Introduction

Renal cell carcinoma (RCC) is the most common malignancy of the kidney, accounting for 5% and 3% of all oncological diagnoses in men and women, respectively (1, 2). Multiple studies have been demonstrated the prognostic and predictive value of various inflammatory markers in RCC patients (3–6). Recent studies have shown that the lymphocyte to C-reactive protein ratio (LCR) may serve as a prognostic marker in gastrointestinal and genitourinary malignancies including colorectal cancer (7–9), gastric cancer (10, 11), hepatocellular carcinoma (12), metastatic prostate cancer (13), and bladder cancer (14). The prognostic value of LCR in RCC remains unexplored. We aimed to examine the clinical utility of LCR for survival outcomes in a large, diverse and international cohort of RCC patients, and to enhance the prognostic stratification of UCLA Integrating Scoring System (UISS), a widely validated prognostic model in RCC (15).

## Methods

### Study population and data collection

We conducted a retrospective multi-institutional analysis of RCC patients with preoperative CRP values and absolute lymphocyte counts utilizing the International Marker Consortium for Renal Cancer (INMARC) database of patients who underwent either partial or radical nephrectomy between June 2009 and December 2025 (3, 16, 17). Each participating center obtained Institutional Review Board (IRB) approval for the collection and management of de-identified patient data, with approvals granted by the respective IRBs of all participating institutions. Given the retrospective use of anonymized data, informed consent was waived in accordance with institutional and regulatory guidelines, and is not applicable for this study. Database managers were responsible for inputting the data into institutional datasets. Collected data included baseline and tumor characteristics, including pathological American Joint Committee on Cancer (AJCC) tumor stage (18) and UCLA Integrated Staging System (UISS) prognostic risk group (15), as well as preoperative CRP level, preoperative absolute lymphocyte count, and survival outcomes. Laboratory results were entered by data managers at the participating centers using standardized units, with CRP recorded in mg/L and absolute lymphocyte count recorded in cells/µL.

### Statistical and machine learning-based analyses

The lymphocyte to CRP ratio (LCR) was calculated as follows: lymphocyte count (cells/μL)/CRP level (mg/L). The LCR cutoff for 5-year cancer-specific mortality (CSM) was determined in the entire study cohort using time-dependent receiver operating characteristics/area under the curve (ROC/AUC) and the Youden index. The Youden index was calculated as sensitivity + specificity −1. Patients were categorized as having ‘low’ or ‘high’ levels of LCR. The differences between low and high LCR groups were analyzed using the Mann–Whitney U test and the Chi-squared test. Cox proportional-hazards regression was performed using the observed time from surgery to death or last follow-up, together with a binary event indicator. Patients who did not experience the outcome were right-censored at their last known follow-up. Utilizing Multivariable Cox regression analysis (MVA), hazard ratio (HR) of each variable was estimated for CSM and all-cause mortality (ACM). LCR, along with the risk factors of the UISS model (tumor stage, ECOG, and nuclear grade), were included in the Cox proportional hazards model to compare their impact on CSM. The 5-year cumulative incidence of RCC-specific mortality between low and high LCR groups were compared using Gray’s test with death from causes other than the outcome of interest treated as a competing event.

As an exploratory analysis, a gradient boosting machine (GBM) model was developed to assess whether incorporating LCR alongside established UISS factors could improve prognostic discrimination. The model included AJCC tumor stage, ECOG performance status, nuclear grade, CCI, and LCR. The cohort was randomly split into training (80%) and validation (20%) sets, using a fixed random seed to ensure reproducibility. Model fitting was performed exclusively on the training set, while all performance metrics were evaluated on the independent validation cohort. Predicted probabilities were generated on the validation cohort and used for calibration assessment. Using Classification and Regression Tree (CART) analysis, LCR was incorporated alongside UISS prognostic risk factors to develop a decision tree for survival risk stratification. Based on relative hazard ratio distributions, a modified UISS model was determined, allowing patients to be reclassified into low-, intermediate-, and high-risk groups. The prognostic performance of UISS and modified UISS models was evaluated using discrimination and calibration metrics in accordance with TRIPOD+AI recommendations (19). The Kaplan-Meier method and the log-rank test were used to compare overall (OS) and cancer-specific survival (CSS). For statistical analyses, Stata18/SE software package (StataCorp, Houston, USA) and R statistical software (version 4.3.2) with ‘survival’ software package were used. In all tests, a 2-sided p < 0.05 was considered significant.

## Results

### Baseline characteristics

A total of 2071 RCC patients were analyzed in this retrospective study (1,415 (68%) male; median follow-up 60 months). In the entire study cohort, the cut-off of LCR for 5-year CSM determined to be 237.87 (AUC=0.81) (**Supplementary Table 1**). Patients were classified into low LCR (LCR<237.87, n=598) and high LCR (LCR ≥237.87, n=1473). Patients with low LCR had higher BMI (p<0.001), higher CCI (<0.001), larger tumor size (p<0.001), and higher nuclear grade (p<0.001) (**Table 1**).

**Table 1:** Baseline characteristics of the study population.

Variable	High LCR N = 1,473*^1^*	Low LCR N = 598*^1^*	p-value*^2^*
Age (years)	62 (52, 69)	61 (53, 70)	0.6
Sex (Male)	1,017 (69%)	398 (67%)	0.3
Ethnicity White Black Asian Other	407 (28%) 188 (13%) 837 (57%) 41 (2.8%)	283 (47%) 128 (21%) 167 (28%) 20 (3.3%)	<0.001
ECOG 0 ≥1	1,273 (86%) 200 (14%)	461 (77%) 137 (23%)	<0.001
BMI	25.1 (22.3, 29.2)	27.0 (23.2, 32.0)	<0.001
Charlson Comorbidity Index	4 (2, 5)	6 (3, 9)	<0.001
Tumor Size	3.5 (2.3, 5.2)	6.5 (3.5, 10.5)	<0.001
Type of Surgery Radical Partial	755 (51%) 718 (49%)	466 (78%) 132 (22%)	<0.001
Positive margins	55 (3.7%)	37 (6.2%)	0.014
Histology Clear cell RCC Non-clear RCC	1,114 (76%) 359 (24%)	453 (76%) 145 (24%)	>0.9
AJCC Tumor Stage 1 2 3 4	1,109 (75%) 55 (3.7%) 235 (16%) 74 (5.0%)	235 (39%) 24 (4.0%) 193 (32%) 146 (24%)	<0.001
Nuclear Grade 1-2 3-4	873 (59%) 600 (41%)	188 (31%) 410 (69%)	<0.001
CRP Level ≥ 5 mg/L < 5 mg/L	135 (9.2%) 1338 (90.8%)	573 (96%) 25 (4%)	<0.001
Absolute Lymphocyte Count (cell/µl)	1670 (1310, 2100)	1425 (1052, 1888)	<0.001
UISS Low Risk Intermediate Risk High Risk	667 (45%) 707 (48%) 99 (6.7%)	113 (19%) 303 (51%) 182 (30%)	<0.001

*^1^*Median (IQR); n (%).

*^2^*Wilcoxon rank sum test; Pearson’s Chi-squared test.

Cut-off point of LCR = 237.87.

### Multivariable analyses

Independent predictors of worsened ACM were increasing age (HR = 1.02, p<0.001), ECOG score ≥1 (HR = 1.38, p=0.015), radical nephrectomy (HR 1.85, p=0.004), higher tumor stage (HR 1.80, p<0.001), nuclear grade 3-4 (HR 1.70, p=0.002), low LCR (HR 2.75, p<0.001). For CSM, ECOG score ≥1 (HR = 1.56, p=0.011), higher tumor stage (HR 3.16, p<0.001), nuclear grade 3-4 (HR 3.02, p<0.001), and low LCR (HR 2.70, p<0.001) were identified as independent predictors (**Table 2**).

**Table 2:** Multivariable cox regression analysis of clinical and pathological factors associated with 5-year all-cause mortality (ACM) and cancer-specific mortality (CSM).

Variable	ACM			CSM		
	HR*^1^*	95% CI*^1^*	p-value	HR*^1^*	95% CI*^1^*	p-value
Age	1.02	1.01, 1.03	<0.001	1.00	0.99, 1.02	0.6
Sex (male vs. female)	1.08	0.84, 1.39	0.6	0.98	0.71, 1.37	>0.9
ECOG (≥1 vs. 0)	1.38	1.06, 1.79	0.015	1.56	1.11, 2.20	0.011
Surgery (radical vs. partial)	1.85	1.22, 2.80	0.004	1.08	0.57, 2.04	0.8
Margins (positive vs. negative)	1.20	0.76, 1.91	0.4	1.40	0.85, 2.33	0.2
Histology (clear vs. non-clear)	0.51	0.39, 0.66	<0.001	0.44	0.31, 0.63	<0.001
AJCC Tumor Stage	1.80	1.60, 2.04	<0.001	3.16	2.58, 3.87	<0.001
Nuclear grade (3–4 vs. 1-2)	1.70	1.22, 2.36	0.002	3.02	1.61, 5.66	<0.001
LCR (Low vs. High)	2.75	2.15, 3.53	<0.001	2.70	1.91, 3.81	<0.001

*^1^*HR, Hazard Ratio; CI, Confidence Interval.

### Predictive performance evaluation using machine learning

A GBM model was developed to predict 5-year CSM. The cohort was randomly divided into a training set (80%; n = 1,664) and an independent validation set (20%; n = 407). In the validation cohort, 5-year CSM rate was 13% (n=53). The model demonstrated good discriminative performance, with an AUC of 0.86 in the validation cohort. The effect of LCR on 5-year CSM among the risk factors of UISS prognostic model was compared utilizing the Cox proportional hazards model in the CART analysis. LCR (HR 2.60, p<0.001), tumor stage (Stage II: HR 6.7, Stage III: HR 6.5, Stage IV: HR 26.8, p<0.001) nuclear grade (HR 3.0, p<0.001) and ECOG (HR 1.6, p=0.002) were included in the Classification and Regression Tree (CART) analysis. The distribution of the relative hazard ratios (RHR) categorized into three risk groups: HR <1.0 (low risk), HR 1.0-2.0 (intermediate risk), HR >2.0 (high risk) (**Figure 1**). Based on the stratification of RHRs, a novel modified UISS prognostic model is developed. The ROC/AUC analysis for predicting 5-year CSM revealed an AUC of 0.91 for the modified UISS model and 0.85 for the classic UISS prognostic model. The AUCs for predicting 5-year ACM in the modified and classic UISS model were 0.82 and 0.75, respectively (**Figure 2**).

**Figure 1:**
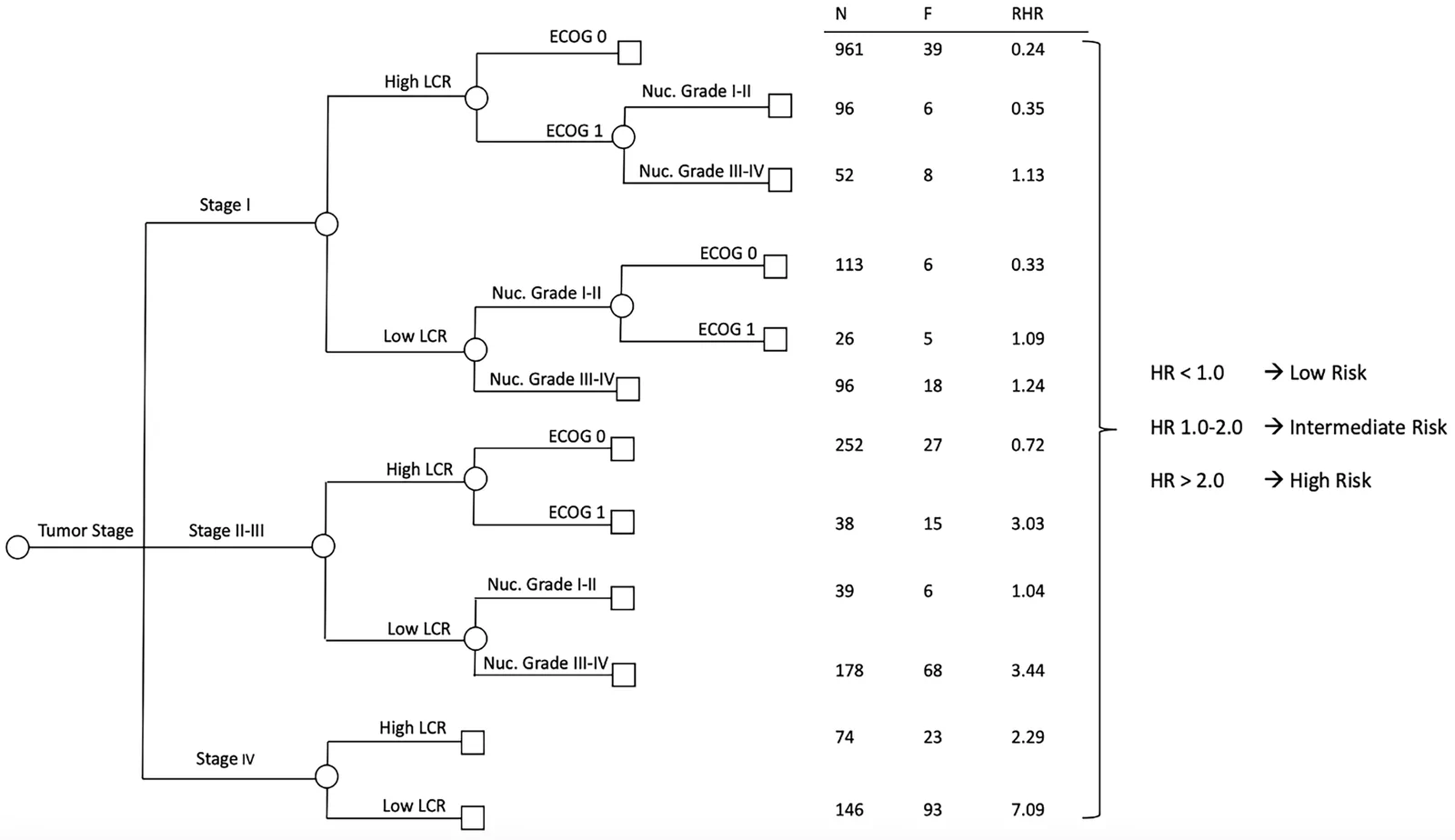
Classification and regression tree (CART) analysis used to reclassify patients into prognostic risk groups according to the distribution of relative hazard ratios (RHRs). Patients were classified as low risk (RHR <1.0), intermediate risk (RHR 1.0–2.0), or high risk (RHR >2.0). ECOG, Eastern Cooperative Oncology Group performance status; F, Number of events; LCR, lymphocyte-to-C-reactive protein ratio; N, Number of patients; RHR, Relative hazard ratio.

**Figure 2:**
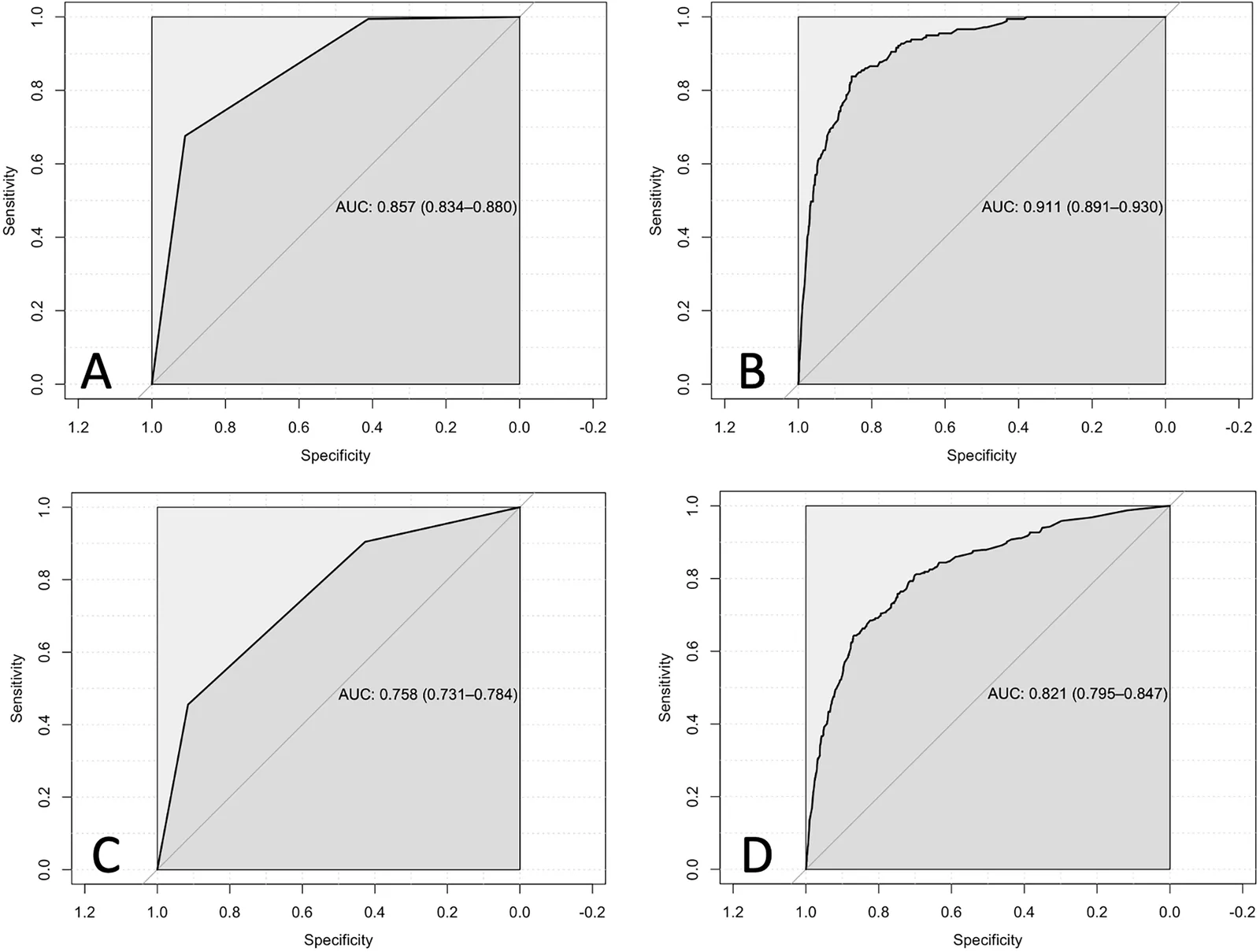
Receiver operating characteristic/area under curves (ROC/AUCs) for predicting 5-year CSM in the standard UISS model **(A)** and in the modified UISS model **(B)**. ROC/AUCs for predicting 5-year ACM in the standard UISS model **(C)** and in the modified UISS model **(D)**. The x-axis displays specificity in decreasing order from 1 to 0, which is mathematically equivalent to displaying 1 − specificity in increasing order. AUC, Area under the curve; ACM, All-cause mortality; CSM: Cancer-specific mortality; LCR, Lymphocyte-to-C-reactive protein ratio; ROC, Receiver operating characteristic; UISS, UCLA Integrated Staging System.

### Kaplan-Meier survival analyses

For low vs. high LCR, OS of 84.6% vs. 94.3% were observed in stage I (p<0.0001), 66.9% vs. 82.1% in stage II (p=0.064), 57.7% vs. 80.4% in stage III (p<0.0001), and 28.3% vs. 64.3% in stage IV (p<0.0001). We noted significant difference of CCS between low vs. high LCR in each tumor stage. 5-year CSS of tumor stages were 96.9% vs. 98.9% in stage I (p=0.015), 73.7% vs. 93.4% in stage II (p=0.0087), 80.6% vs. 87.5% in stage III (p=0.014), and 32.2% vs. 69.2% in stage IV (p<0.0001), respectively (**Figures 3**, **4**).

**Figure 3:**
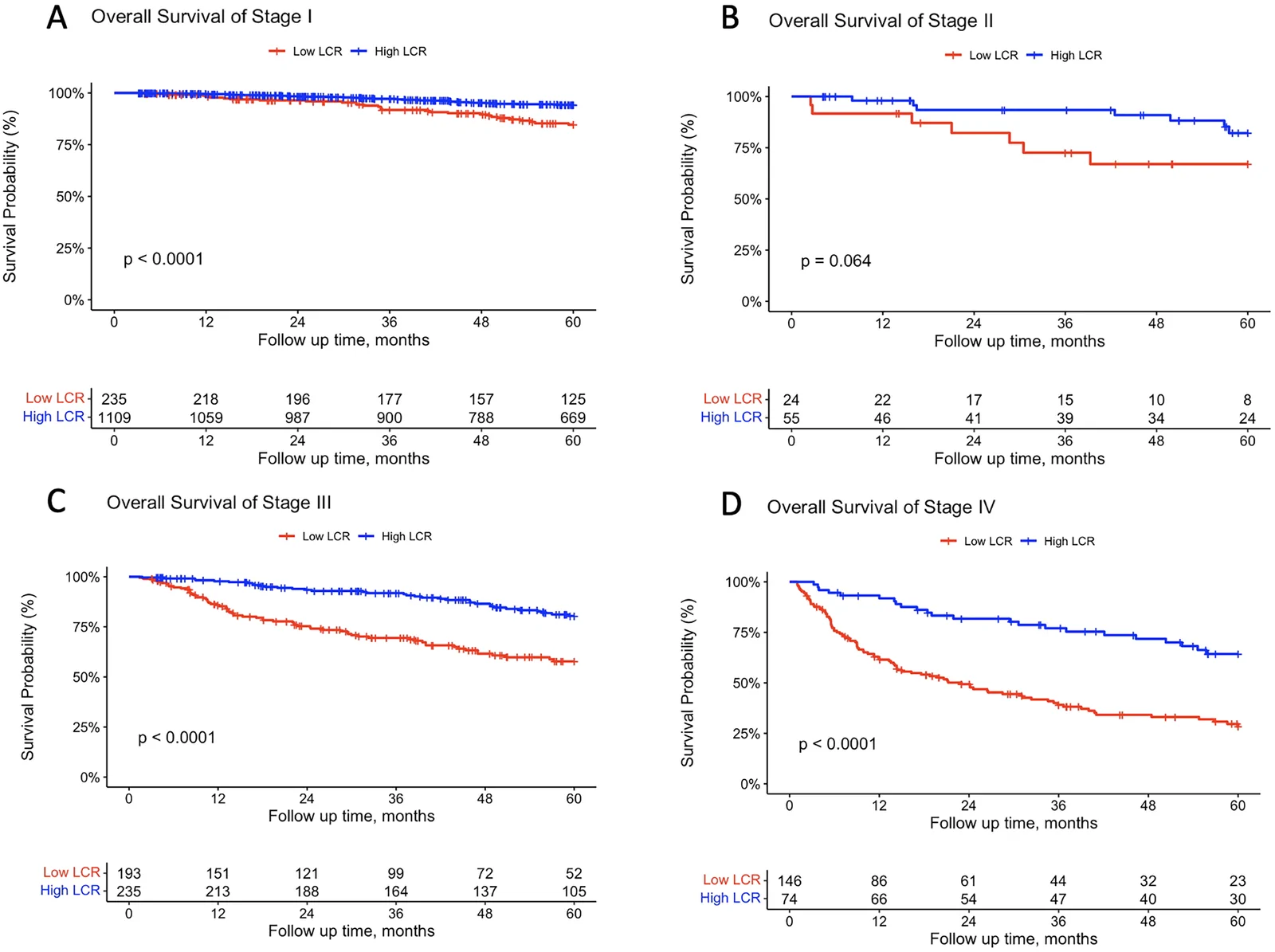
Kaplan-Meier Survival Analyses of OS comparing low and high LCR in Stage I **(A)**, Stage II **(B)**, Stage III **(C)**, Stage IV **(D)**. OS, Overall survival.

**Figure 4:**
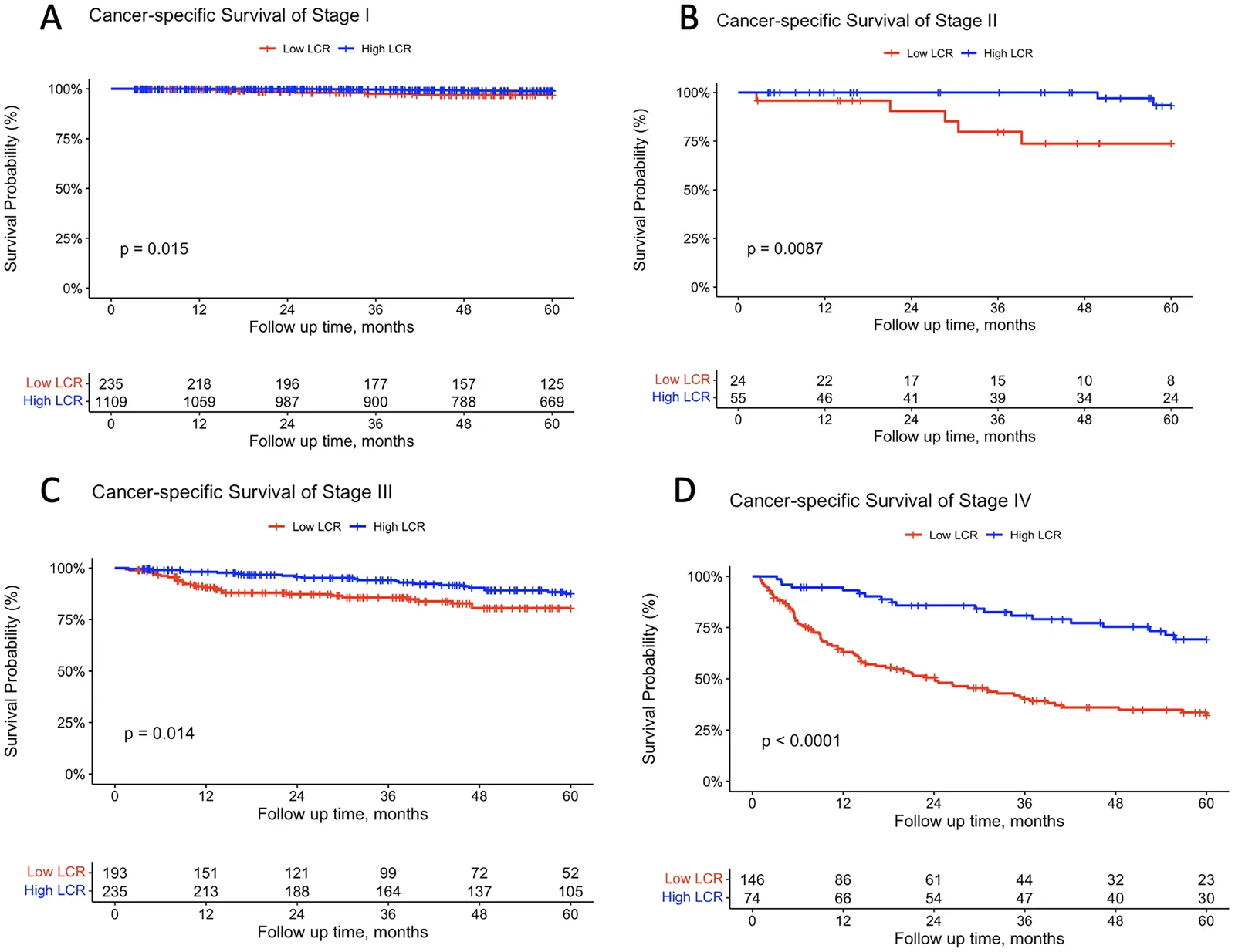
Kaplan-Meier Survival Analyses of CSS comparing low vs. high LCR in Stage I **(A)**, Stage II **(B)**, Stage III **(C)**, Stage IV **(D)**. CSS, Cancer-specific survival.

For the classic UISS model, OS of the risk groups were 95.3% for low-risk, 82.6% for intermediate-risk, 44.2% for high-risk, while CSS were observed as follows: 99.8% for low-risk, 92.7% for intermediate-risk, and 51.4% for high-risk. For the modified UISS model, KMA revealed OS rates of 91.3%, 64.3%, and 42.3% for low-, intermediate-, and high-risk, respectively. CSS was noted as 97.5% for low-risk, 69.2% for intermediate-risk, 55.6% for high-risk (**Figure 5**).

**Figure 5:**
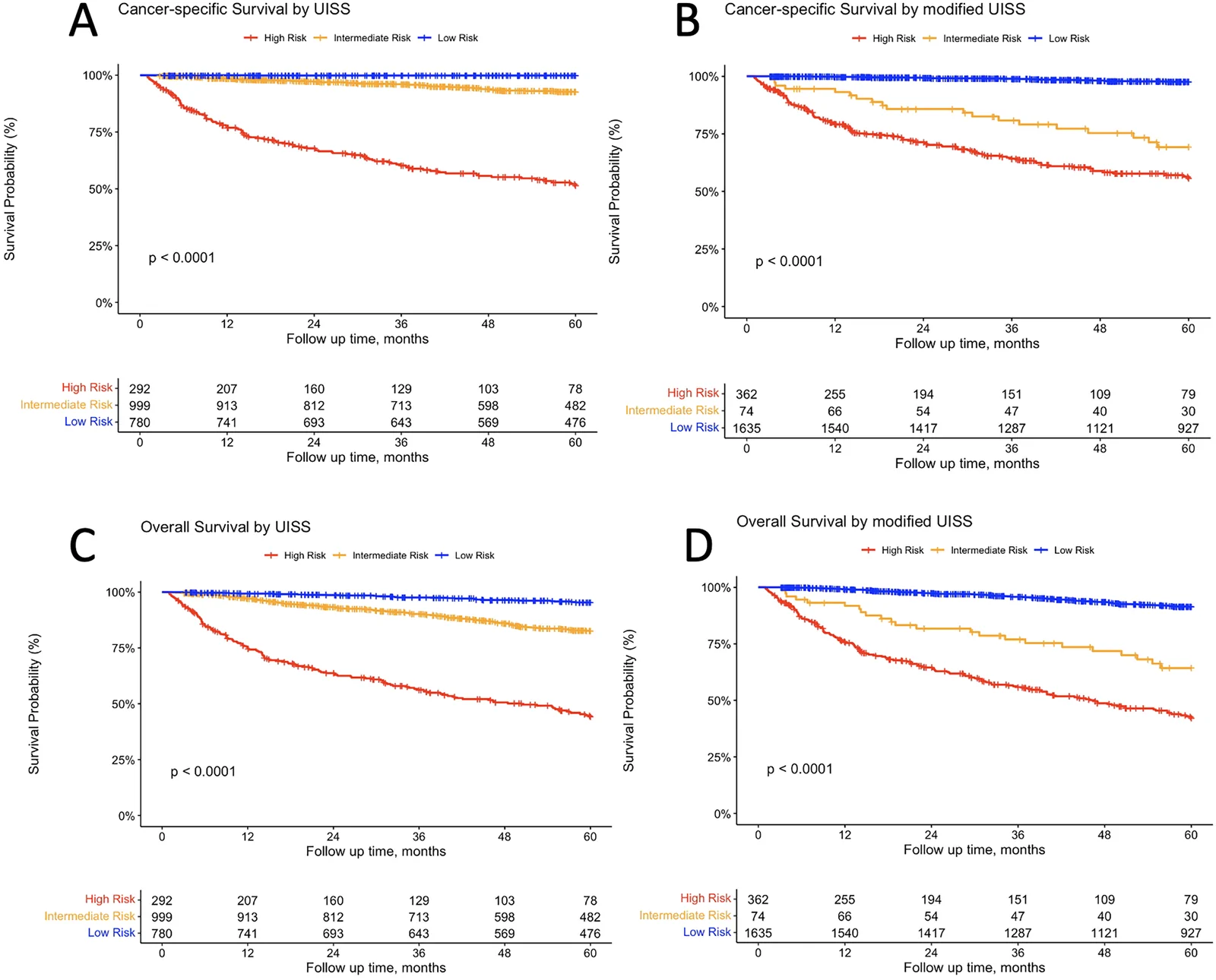
Kaplan–Meier survival curves comparing risk-group stratification using the standard and modified UISS models. Cancer-specific survival is presented for the standard UISS model **(A)** and modified UISS model **(B)**, while overall survival is presented for the standard UISS model **(C)** and modified UISS model **(D)**. CSS, cancer-specific survival; KMA, Kaplan–Meier analysis; OS, overall survival; UISS, UCLA Integrated Staging System.

### Competing-risk analysis

The five-year cumulative incidence of RCC-specific mortality was 23.7% (95% CI: 20.0%–27.6%) in the low-LCR group and 4.3% (95% CI: 3.2%–5.5%) in the high-LCR group (p<0.0001). The corresponding cumulative incidence of other-cause mortality was 14.2% (95% CI: 11.1%–17.4%) and 5.1% (95% CI: 3.9%–6.3%), respectively (**Supplementary Table 2**).

## Discussion

This study displays the clinical significance of LCR in a large international multicenter RCC cohort. First, low LCR was an independent predictor for ACM and CSM and associated with unfavorable OS and CSS. Second, incorporating LCR into a machine-learning based analysis resulted in an enhanced prognostication for 5-years cancer-specific survival. Our findings indicate that LCR could serve as a prognostic marker in RCC patients, which is easy-to-implement in preoperative oncological assessment to improve risk stratification.

Clinical significance of CRP in RCC patients has been investigated in previous studies. In a cohort of 2445 RCC patients, elevated CRP (> 5.0 mg/L) was an independent predictor for disease recurrence (HR 1.85, p=0.005) and all-cause mortality (HR = 4.13, p<0.001) and worsened OS was observed for all stages when comparing normal to elevated CRP: 5-year OS of 98% vs 81% (p=0.001), 94% vs 80% (p=0.103), 94% vs 65% (p=0.001), 99% vs 38% (p<0.001) for stages 1, 2, 3, and 4, respectively (3). In a meta-analysis of 24 studies, elevated CRP was associated with higher stage (HR = 2.90, p<0.00001) and higher grade (HR = 4.31, p<0.00001), and poorer OS (HR = 1.51, p<0.00001) and CSS (HR = 3.91, p<0.00001) (20). Furthermore, Ueda et al. have reported that pretreatment low absolute lymphocyte count was an independent risk factor for worsened PFS (HR = 2.3, p=0.046) and OS (HR = 4.1, p=0.034) for patients with metastatic RCC treated with Nivolumab (21). These findings suggest a systemic inflammatory response in RCC, as reflected by alterations in lymphocyte count and CRP level and underscore their prognostic utility in survival outcomes.

MVA revealed that low LCR was an independent predictor of worsened ACM (HR = 2.75, p<0.001) and CSS (HR = 2.70, p<0.001). Our results represent the initial clinical significance of LCR in RCC and are consistent with previous studies in other malignancies. In a meta-analysis of 21 cohort studies in gastrointestinal malignancies involving 9,131 patients, lower LCR levels were associated with poorer survival outcomes (p<0.001), supporting its role as a prognostic marker (9). Similar results have been observed in genitourinary malignancies. In a cohort of 361 metastatic prostate cancer patients, low LCR was correlated with poor OS compared to high LCR (48.5% vs 78.6%, p<0.001) and LCR was an independent predictor for OS (HR = 2.27, p=0.003) (13). In a retrospective study of 201 patients who treated with radical cystectomy, high LCR group was independently associated with improved OS (HR = 0.41, p<0.001) and disease-free survival (HR = 0.40, p<0.001) (14). We analyzed 5-year OS and CSS comparing low vs. high LCR in each tumor stage. Across tumor stages for low vs. high LCR, lowest difference in OS of 84.6% vs. 94.3% (p<0.0001) and CSS of 96.9% vs. 98.9% (p=0.015) were observed in stage I, whereas the largest difference noted in stage IV for OS of 28.3% vs. 64.3% (p<0.0001) and CSS of 32.2% vs. 69.2% (p<0.0001) (**Figures 3**, **4**). To our knowledge, this study is the first to demonstrate an independent association between LCR and survival outcomes in RCC patients. Further investigations are needed to assesses the mechanism between inflammatory-based blood parameters and tumor stage-related survival outcomes of RCC patients.

A major challenge of the UISS prognostic model lies in providing optimal differentiation of intermediate risk group patients, the largest group of the model, separating them from low- and high-risk groups (22–24). Several studies have focused on improved prognostication of RCC patients compared to UISS. In a study cohort of 240 patients, Kroeger et al. demonstrated a number of pathological and genetic markers, enhancing the predictability of disease recurrence for the high-risk patients from 0.68 to 0.73 (23). In a study of 290 RCC patients, a scoring model based on CRP and the TNM classification was validated with a concordance index of 0.86 (24). Lamb et al. demonstrated an equivalent predictability of the Glasgow Prognostic Score, based on CRP and albumin, with an AUC of 0.80 in a cohort of 169 RCC patients when compared to the UISS, which showed an AUC of 0.77 (25, 26). In this study, we conducted internal validation utilizing GBM model, which demonstrated good discriminative ability, with an AUC of 0.86, providing robust prediction of 5-year CSM. Furthermore, our results in CART analysis present the enhanced predictability achieved by incorporating LCR into the UISS prognostic model, with an AUC of 0.91 compared to an AUC of 0.85 for the classic UISS model. Moreover, this study displays better stratification of risk groups, with CSS of 97.5% for low-risk, 69.2% for intermediate-risk, 55.6% for high-risk, while we observed CSS of 99.8% for low-risk, 92.7% for intermediate-risk, and 51.4% for high-risk in the standard UISS model. Our machine learning-based results demonstrate the initial prognostic significance of LCR alongside established risk factors, including TNM staging, nuclear grade, and ECOG. Overall, TNM staging emerged as the primary determinant factor and LCR served as the secondary factor of the decision tree in the CART analysis. For Stage IV, LCR was the only factor to stratify survival risk. In Stages II and III, ECOG was the decision-making factor for patients with high LCR, while nuclear grade played the determinant role for patients with low LCR in the re-stratification of patients into risk groups. For Stage I, primary factor was LCR, followed by ECOG and nuclear grade. Taken together, our findings demonstrate the utility of LCR in stratifying survival risk in RCC, leading to a significant improvement in prognostication when added to the UISS model. Indeed, while external validation is requisite, our analysis represents a significant refinement of the classic UISS prognostic model, demonstrating promising clinical utility of LCR for improving preoperative risk stratification in RCC patients.

Although our results and their clinical utility are supported by the diversity and size of our international cohort, the retrospective design of this study and its inherent biases significantly impact its limitations. The inclusion of only patients who underwent nephrectomy may limit the generalizability of our findings, particularly for stage IV disease. Changes in systemic treatment during the study period may also have influenced survival outcomes. Moreover, limitations of the machine learning–based analyses should be acknowledged (27). The model underwent internal validation only. Therefore, the AUC should be considered an exploratory estimate of internal discrimination, and external validation using independent datasets is required to confirm our findings. Dichotomizing LCR into low- and high-risk groups may have limited the assessment of the relationship between LCR and mortality. The interval between preoperative laboratory assessment and surgery was not consistently available, and acute infections, inflammatory conditions, or other factors affecting CRP and lymphocyte levels were not uniformly captured. Residual confounding from BMI, comorbidity burden, ethnicity, tumor size, and other measured and unmeasured factors cannot be excluded. Differences in patient selection, clinical practice, and laboratory methods among the participating centers may therefore have influenced the findings. Indeed, while our findings are constrained by several limitations, this study stands as a first clinical assessment of LCR in RCC. It highlights the clinical utility of LCR as a simple ratio based on common blood parameter, refining the well-known UISS prognostic model to enhance the predictive accuracy for survival outcomes.

## Conclusion

Our findings demonstrate the clinical utility of LCR for improving prognostic stratification in RCC patients. Low LCR was associated with worsened cancer-specific and overall survival rates. Inclusion of LCR in the UISS prognostic model enhanced prognostic performance and improved survival risk stratification. However, these findings are exploratory and require external validation before clinical application.

## Data Availability

The data analyzed in this study is subject to the following licenses/restrictions: Data were obtained from the International Marker Consortium for Renal Cancer (INMARC) database, a multicenter international registry database. Due to institutional review board requirements and data-sharing agreements, the dataset is not publicly available. Requests to access these datasets should be directed to Ithaar Derweesh, iderweesh@gmail.com.
