# Supplementary material for: Lymphocyte to C-reactive protein ratio and improved prognostication of renal cell carcinoma: machine learning-based analysis
Source: Front Oncol. 2026 Oct 13;16:1907942. doi: 10.3389/fonc.2026.1907942 (PMC13710031; doi:10.3389/fonc.2026.1907942)
Supplement: Supplementary file 1 [file Table1.docx]

**Supplementary Table 1:** Diagnostic performance of the LCR cut-off for five-year cancer-specific mortality.

| Methods | **LCR Cut-off** | **AUC** | **Sensitivity, %** | **Specificity, %** | **Youden index** |
| --- | --- | --- | --- | --- | --- |
| Censoring-adjusted time-dependent ROC analysis | 237.87 | 0.81 (0.783–0.854) | 67.86 (60.63–74.90) | 80.00 (77.60–82.39) | 0.479 |
| LCR: Lymphocyte to CRP ratio; AUC: Area under the curve; ROC: receiver operating characteristics | | | | | |

**Supplementary Table 2:** Five-year cumulative incidence of RCC-specific and other-cause mortality according to LCR group

| **Outcome** | **Low LCR, n = 598** | **High LCR, n = 1,473** | **Absolute difference** | **p value** |
| --- | --- | --- | --- | --- |
| RCC-specific mortality | 23.7% (20.0%–27.6%) | 4.3% (3.2%–5.5%) | 19.4 | <0.0001 |
| Other-cause mortality | 14.2% (11.1%–17.4%) | 5.1% (3.9%–6.3%) | 9.1 | — |
| RCC: Renal cell carcinoma; LCR: Lymphocyte to CRP ratio | | | | |
